# Understanding Vernal Keratoconjunctivitis: Beyond Allergic Mechanisms

**DOI:** 10.3390/life11101012

**Published:** 2021-09-26

**Authors:** Marta Sacchetti, Rocco Plateroti, Alice Bruscolini, Rosalia Giustolisi, Marco Marenco

**Affiliations:** Department of Sense Organs, University Sapienza of Rome, Viale del Policlinico, 155, 00161 Rome, Italy; rocco.plateroti@uniroma1.it (R.P.); alice.bruscolini@uniroma1.it (A.B.); rosalia.giustolosi@uniroma1.it (R.G.); marco.marenco@uniroma1.it (M.M.)

**Keywords:** vernal keratoconjunctivitis, allergic disease, sex hormones, innate immunity, neuroinflammation

## Abstract

Vernal keratoconjunctivitis (VKC) is a chronic, recurrent, inflammatory disease of the cornea and conjunctiva mostly affecting boys in prepubertal age. VKC recurrence is characterized by intense symptoms of itching, redness, and photophobia associated with corneal damage, impairment of visual function, and quality of life. The pathogenesis of VKC has not yet been completely understood, and it is still controversial. In fact, VKC is considered an ocular allergic disease due to the involvement of immunoglobulin E, eosinophils, and mast cells, and of a lymphocyte T-helper type 2 reaction. However, approximately half of VKC patients have negative allergological history and testing, suggesting that other pathogenic mechanisms participate in VKC development and severity. Specifically, evidence suggests that genetic, endocrine, neuronal factors and an imbalance of innate immunity are involved in the pathogenesis of VKC. The purpose of this review is to summarize evidence on the pathogenic role of innate immunity, neuroimmune reaction, and hormonal changes in VKC. Increasing understanding of the pathogenic mechanisms behind VKC may lead to the identification of novel biomarkers for diagnosis and/or potential therapeutic targets in order to improve the management of this challenging condition.

## 1. Introduction

Vernal keratoconjunctivitis (VKC) is a rare, sight-threatening, chronic, inflammatory disease of the cornea and conjunctiva characterized by recurrent flare-ups of ocular surface inflammation, causing intense ocular symptoms of itching, redness, and photophobia associated with corneal damage and impairment of visual function and quality of life [1,2,3].

VKC is a clinical form of allergic conjunctivitis diseases, together with seasonal and perennial allergic conjunctivitis (AC) and atopic keratoconjunctivitis (AKC) [4]. In fact, VKC shares some clinical features and pathogenic mechanisms with other forms of AC, including ocular itching, swelling, redness, and conjunctival papillary reaction associated with immunoglobulin (Ig) E-mediated release of histamine and other allergic reaction mediators from mast cells [4]. However, this is likely not the only mechanism involved in VKC immunopathogenesis, as only 50% of cases of VKC show allergic sensitization [4]. Several studies have demonstrated that the inflammatory reaction occurring in VKC also involves a lymphocyte T-helper (Th) type 2-driven reaction, a late-phase allergic reaction with eosinophil infiltration, and extracellular matrix remodeling [1,2]. In addition, the demographical, geographical, and clinical characteristics of VKC suggest that other endocrine, environmental, and/or genetic factors may play a role in the pathogenesis of this challenging condition.

Specifically, VKC mostly affects children, with higher propensity in boys than girls, and, in most cases, spontaneously resolves after puberty, suggesting that an imbalance of sex hormones may play a role in its pathogenesis [2,3,5]. A higher prevalence of the condition is observed in warm regions, such as the Mediterranean area, Central and South America, Japan, Central and West Africa, and the Middle East, which also suggests a potential pathogenic role of genetic and/or environmental factors [2,3]. Finally, recent studies point to a possible role of innate immunity, including toll-like receptors (TLRs) and natural killer (NK) cells, in the development and severity of VKC [6,7,8,9].

Increasing understanding of the pathogenic mechanisms associated with the onset and severity of VKC may lead to the identification of novel therapeutic targets and potentially improve the management of patients. The aim of this review is to describe the most recent advancements in the knowledge of VKC pathogenesis, including the role of innate immunity, neuroinflammatory reaction, and hormones.

## 2. Clinical Characteristics of VKC

Vernal keratoconjunctivitis is a bilateral, recurrent, inflammatory disease of the ocular surface, most frequently observed in boys of prepubertal age [1]. In most cases, VKC spontaneously resolves after puberty. However, an adult form has also been described [10,11]. Different studies reported that nearly 50% of patients with VKC have atopic sensitization, and approximately 40% show atopic associated conditions, such as asthma, allergic rhinitis, and eczema [2,3,12,13].

**Figure 1:**
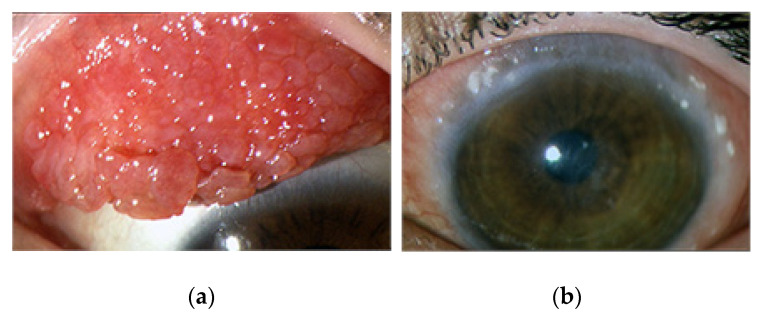
(**a**) Tarsal VKC with upper tarsal giant papillary reaction, (**b**) limbal VKC with limbal papillary reaction and the so-called Horner-Trantas dots.

A clinical characteristic sign of VKC is the presence of a giant conjunctival papillary reaction of the upper tarsal conjunctiva and/or limbal region resulting from chronic inflammation and extracellular matrix remodeling [1,14,15,16]. Specifically, tarsal VKC is characterized by giant hypertrophic papillae at the upper tarsal conjunctiva with a cobblestone appearance, while the limbal form is characterized by gelatinous infiltration around the cornea. Mixed forms show both tarsal and limbal papillary reactions [16] (Figure 1a,b).

**Figure 2:**
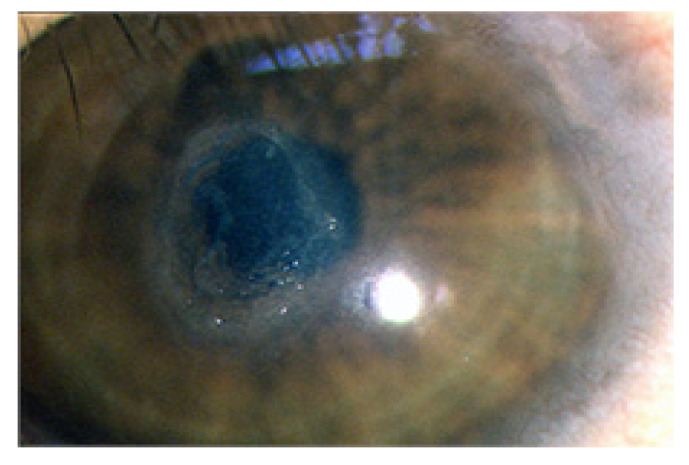
Corneal epithelial defect in a patient with VKC.

Patients with VKC experience recurrent flare-ups of ocular surface inflammation, most often during the spring–summer season. However, in severe cases, the cycle is ongoing throughout the year [1,16]. The associated symptoms are intense itching, photophobia, and tearing. During recurrences, they show severe conjunctival hyperemia, swelling, thick mucus discharge, and corneal damage [2,16,17,18]. Corneal involvement in VKC is the consequence of intense ocular surface inflammation, causing the development of superficial punctuate keratopathy, which may progress to corneal epithelial defects and shield ulcers [14,17,19] (Figure 2).

Corneal ulcers are reported in 3%–11% of cases and may cause pain and impairment of visual function [19]. Horner-Trantas dots surrounding the cornea represent a typical sign of active inflammation and are mostly due to eosinophil infiltration (Figure 1b). More severe cases, with long-standing disease, may develop corneal neovascularization and scarring, associated with permanent impairment of visual function [2,17,19].

Morphological alterations of corneal epithelium, stroma, and nerves have been observed in patients with VKC by performing corneal in vivo confocal microscopy, suggesting a corneal neuropathic involvement in VKC [20,21]. It is worthy to note that corneal nerves not only provide corneal sensitivity but, by releasing neuropeptides, are also fundamental to supply trophic support to corneal epithelial and stromal cells [22]. Therefore, the decreased density and number of corneal nerve fibers may contribute to the development of corneal epithelial alterations in VKC [21].

The intense ocular discomfort, associated with the chronic and recurrent clinical course of the disease, the need for frequent visits, and long-term topical treatments, significantly impairs the quality of life and social functioning of children with VKC and their caregivers [23,24].

Management of patients with VKC represents a challenge for ophthalmologists. In fact, antiallergic eye drops such as antihistamines and/or mast cell stabilizers are effective only in very mild cases. The majority of patients in the active phase of ocular inflammation require the use of topical steroids and/or other immunosuppressive drugs [16,25,26]. Topical steroids are very effective in controlling the signs and symptoms of active disease. However, their chronic use is associated with the development of severe ocular complications, such as glaucoma and cataract [1,2,27]. Therefore, steroid sparing agents, such as topical cyclosporine A (CsA) or topical tacrolimus (FK506), are currently used for the chronic treatment of VKC [28,29,30].

A correct therapeutic approach to patients with VKC should be based on several considerations, including clinical severity, the long-term course of the disease, and a careful risk/benefit evaluation. Several clinical grading systems of VKC have been proposed in order to standardize the management of cases and improve clinical outcomes. Currently, the best-known grading scale is the Bonini scale [16,25,31,32]. This clinical grading system includes an evaluation of the severity of symptoms, conjunctival hyperemia, and papillary reaction, as well as of corneal involvement, including Horner-Trantas dots and epithelial damage [16]. Bonini et al. also proposed a therapeutic approach for VKC patients based on this severity grading system. Specifically, patients in the quiescent phase (grade 0) do not require treatment until symptoms occur. Patients with mild to moderate VKC (grades 1 and 2) may be treated with occasional or daily use of topical antiallergic drugs, while in the presence of more severe ocular inflammation associated with corneal damage (grades 3 and 4), the use of topical CsA and/or pulsed high-dose topical steroids is recommended [16]. It is very important to highlight that corneal damage in VKC represents a major concern for ophthalmologists, as it may lead to impairment of visual function [17]. Recently, a new scoring system, the VKC-Collaborative Longitudinal Evaluation of Keratoconus study (VKC-CLEK), has been proposed for the assessment of ocular surface staining in VKC [32]. The VKC-CLEK scheme includes an evaluation of the epithelial damage in both the limbal and perilimbal areas, as well as in the central cornea, to better evaluate and standardize the severity of corneal damage in both limbal and tarsal VKC [32].

Despite efforts to standardize VKC grading and management, this condition, orphan of a specific pathogenic treatment and with an uncertain clinical outcome, remains a challenge for physicians.

## 3. IgE-Mediated Hypersensitivity Reaction in VKC

Some clinical and experimental evidence shows that IgE-mediated hypersensitivity reaction plays a major role in the pathogenesis of VKC [1]. Type I, IgE-dependent, hypersensitivity is characterized by IgE overproduction by immune cells in response to environmental allergens, such as pollens or dust mites. IgE antibodies, by binding to mast cells and basophils, trigger the release of vasoactive mediators [33]. Conjunctival mast cells’ release of inflammatory mediators, including histamine and tryptase, induces the early-phase reaction symptoms of itching, redness, and swelling [34,35]. High levels of both histamine and tryptase have been described in VKC and are associated with increased conjunctival expression of histamine receptors compared with healthy nonallergic subjects [34,35,36]. In addition, patients with VKC show hyper-responsiveness to the histamine conjunctival provocation test, suggesting that a nonspecific conjunctival hyper-reactivity reaction may contribute to the pathogenesis of VKC [37]. Conjunctival mast cells in VKC also release newly formed mediators, such as interferon (IFN), tumor necrosis factor (TNF)-alpha, granulocyte-macrophage colony-stimulating factor, prostaglandins, and leukotrienes, which induce eosinophil and basophil recruitment [34,35,37].

A pathogenic role of IgE-mediated allergic reaction to environmental agents in VKC is also supported by clinical and histopathological studies. Specifically, most patients with VKC have a family history of atopy and/or are affected by other atopic conditions, including asthma, rhinitis, and eczema [2]. In addition, VKC often shows a seasonal course, with onset and flare-ups frequently occurring during dry and hot spring–summer seasons, which are characterized by high pollen count [2,15]. Moreover, approximately 50% of patients with VKC show IgE sensitization, assessed by skin prick test and/or serum-specific IgE measurement [2,15,38]. High levels of specific IgE have been found in the tears and serum of patients with VKC [5,14,39,40,41,42]. Some studies also demonstrated that specific IgE may be found in tears of VKC patients with negative systemic allergometric tests, suggesting that a conjunctival sensitization, with local production of specific antibodies, may occur [15,43]. This hypothesis is supported by evidence of the presence of B lymphocytes expressing IgE in conjunctival lymphoid follicles in VKC specimens [5,42]. A key role of local, Th2-driven, conjunctival overproduction of IgE in VKC has also been shown with evidence of significant increase in CD4 T-helper type 2 (Th2) lymphocytes in the conjunctiva of patients with VKC, associated with increased levels of Th2 cytokines, including IL-4, IL-5, IL-13, and interferon (IFN)-gamma, which stimulate B cell switching to produce IgE [15,44,45,46,47]. Activated Th2 lymphocytes also participate in the recruitment and activation of mast cells and eosinophils [5,14,39,40,41,42,47].

## 4. Cellular Allergic Reaction in VKC

Vernal keratoconjunctivitis is traditionally considered a clinical form of allergic conjunctivitis, in which IgE-mediated hypersensitivity mechanisms play an essential role. Nevertheless, clinical evidence from several studies shows that cellular reaction is also involved in the pathogenesis of VKC [1]. At first, approximately 50% of patients with VKC have a negative allergy test, suggesting that other, non-IgE mechanisms contribute to the inflammatory reaction in VKC [1,33]. In addition, it has been demonstrated that, different from other AC, VKC is characterized by the involvement of the late-phase inflammatory reaction [48]. Conjunctival inflammatory infiltrates in VKC are characterized by the presence of lymphocytes, eosinophils, mast cells, basophils, dendritic cells (DC), and macrophages [15]. An increased number of Th2 lymphocytes was described in tears and conjunctiva of VKC patients associated with Th2-type cytokines, such as IL-4, IL-5, and IL-13 [15,44,47]. A recent paper described an increase in the conjunctival expression of thymic stromal lymphopoietin (TSLP) in VKC when compared with healthy subjects. TSLP is a novel allergy-related cytokine, released by epithelial and mast cells, which activates DCs to induce CD4+T cell differentiation into Th2 and stimulate Th2 allergic inflammation [49,50].

Basophils also exert effector functions in the development and maintenance of Th2-cytokine-mediated inflammation [51]. Basophils express IgE receptors and, upon allergen challenge, release several immunoregulatory and effector mediators, including IL-4, IL-13, histamine, and leukotrienes. IL-13 stimulates goblet cell mucin production and, in synergy with IL-4, promotes isotype switching to IgE in B cell and eosinophil recruitment. Basophils also release IL-8 and CCL5 (RANTES), which induce the recruitment of neutrophils, eosinophils, and macrophages [51].

Eosinophils play a major role in the pathogenesis of VKC. The presence of eosinophils in the conjunctiva of active VKC may be considered a hallmark of the disease [52]. Conjunctival biopsies of VKC patients show an increase in eosinophil infiltration associated with increased tear levels of eosinophil cationic protein (ECP) and major basic protein (MBP) [15,53]. Since eosinophils are absent in the healthy conjunctiva, the presence of eosinophils in conjunctival cytology has been proposed as a marker to confirm the diagnosis of VKC and to monitor response to treatments [54,55]. Eosinophil recruitment in VKC has been associated with increased expression of chemokines by both immune and resident cells, including epithelial cells, vascular endothelial cells, and fibroblasts [15]. Specifically, an increased expression of eotaxin 1 and 2 of monocyte chemoattractant protein (MCP)-1, MCP-3, and RANTES has been described in the tears and conjunctiva of patients with active VKC [56,57]. Activated eosinophils release cytotoxic proteins, such as MBP and ECP, as well as chemokines, collagenases, leukotrienes, and neurotoxins, which exert toxic effects to corneal epithelium and have been associated with corneal damage in VKC [52,53,58,59]. Furthermore, higher tear ECP concentration has been significantly correlated with the clinical severity of VKC [53,60].

## 5. Role of Innate Immunity in VKC

All of the structures of the ocular surface, including the eyelid, lacrimal gland, tear film, cornea, and conjunctiva, provide a first-line defense to the eye. Particularly, corneal and conjunctival epithelia directly interact with the environment and act as a barrier against pathogens, toxic stimuli, and allergens by inducing a defensive response involving both resident and immune cells. In fact, increasing evidence demonstrates that corneal and conjunctival mucosal epithelia play an active role in the ocular surface inflammatory reaction and in stimulating innate immunity [7,61]. Ocular surface epithelia release cytokines, prostaglandins, leukotrienes, and growth factors in response to inflammatory mediators [62]. Specifically, histological studies have shown an increased expression of adhesion molecules, such as intercellular adhesion molecules (ICAM-1) and chemokines on the epithelial cells of VKC, which contribute to the recruitment of inflammatory cells [63]. In addition, changes of the conjunctival epithelial expression of heat shock protein (Hsp) chaperones have been found in VKC patients when compared with healthy controls. Cultured conjunctival cells have also shown changes in the Hsp patterns in response to inflammatory stimuli. These findings suggest that interaction between the chaperoning and the immune systems may influence the progression of VKC [64].

Interestingly, conjunctival epithelium plays a key role in the innate immunity response through the expression of various membrane receptors, such as toll-like receptors (TLRs). TLRs are a family of pattern recognition receptors expressed by resident and immune cells, which recognize microbial compounds and stimulate appropriate adaptive immune reaction [65,66,67,68,69]. In fact, activation of different TLRs induces a specific expression of cytokines and costimulatory molecules that influence the adaptive immune response by inducing T-helper lymphocyte differentiation to the Th type 1 or Th type 2 response [61]. Alteration of mucosal innate immunity may represent a pathogenic mechanism for ocular surface immune disorders, including VKC [7]. Specifically, changes in the conjunctival expression of TLR4 and TLR9 has been demonstrated in patients with VKC when compared with healthy controls, suggesting that TLRs may play a role in the pathogenesis of VKC [9]. A subsequent study also reported that patients with VKC showed improvement in the signs and symptoms after 4 weeks of treatment with *Lactobacillus acidophilus* eye drops, in association with downregulation of both ICAM-1 and TLR4 [70]. Recently, conjunctival gene overexpression of pattern recognition receptors (PRRs) in VKC patients was described, including chemokines, proinflammatory cytokines, and TLR4 and TLR8, suggesting a role of host–pathogen interaction in VKC [71].

A role of innate immunity in VKC pathogenesis has also been supported by the evidence of a significant increase in natural killer (NK) cells infiltrating the conjunctiva of VKC patients when compared with healthy subjects [6]. NK cells are lymphocytes with cytotoxic activity able to destroy tumor cells and virus-infected cells. In addition, NK cells may be induced to release Th2 cytokines, such as IL-4, IL-5, and IL-13, thus influencing immune reaction to a Th2 response. The finding of a significant increase in NK cells in conjunctival tissue of VKC patients supports the hypothesis that a subpopulation of NK cells can influence the Th1/Th2 lymphocyte ratio and thus participate in VKC pathogenesis.

Recently, increased serum levels of high-mobility group box protein (HMGB)-1 have been described in children with VKC [72]. HMGB1 induces inflammatory reaction through binding with the receptor for advanced glycation end products (RAGE), as well as with TLR4 and TLR9 [72]. A subsequent study also demonstrated an increase in tear levels of HMGB1 in VKC patients [73]. These findings suggest a potential role of this protein in the recruitment and survival of eosinophils in VKC.

Increasing understanding of the role of innate immunity, TLRs, and HMGB1 in allergic diseases may lead to the identification of novel potential targets able to modulate immune responses and to reduce ocular surface inflammation.

## 6. Genetic Factors

A genetic influence for the development of allergic diseases is demonstrated by the observation of an increased prevalence of atopic diseases in families with atopic relatives and in monozygotic versus dizygotic twins. An involvement of multiple genes has been demonstrated in the pathogenesis of allergic asthma, rhinitis, eczema, and conjunctivitis. Different genes regulate the presence of increased levels of serum total IgE and specific IgE [74]. Replicated linkage is reported in different atopic phenotypes for total serum IgE (chromosome 11, 4–7) and skin prick tests (chromosome 11), but not for specific IgE. It is possible to hypothesize that different combinations of genes provoke different allergic disease manifestations and expressions. In fact, human chromosomes 5, 6, 11, 12, and 14 have been suggested to be involved in atopic diseases, such as asthma and eczema, while seasonal allergic conjunctivitis susceptibility has been associated with genes on human chromosomes 16, 17, 5, and, to a lesser extent, 6 [75].

The demographical and geographical characteristics of VKC suggest that both genetic and environmental factors may contribute to VKC pathogenesis. Currently, little data have been published on the association between specific HLA genes with VKC [76]. A potential association between VKC and the cytokine gene cluster on chromosome 5q has been hypothesized; however, it has not been confirmed yet [77].

An Italian study on 32 children with VKC reported an association between VKC and HLA class II allele [78]. Specifically, this study showed that specific HLA-DQB1 alleles may contribute to the susceptibility to VKC, with a higher frequency of DQB1*05 in VKC patients when compared with healthy controls. [78] Recently, results of an HLA analysis on monozygotic twins and their father affected by VKC and all family members confirmed the association between HLA alleles DQB1*05:01:01 and HLA-DRB1*01:01:01 with VKC [79,80].

Currently, few candidate atopic genes have been identified. Increasing discovery in this field will clarify the pathophysiological mechanisms of VKC, allowing improved diagnosis timing, prevention of susceptible subjects, and personalized treatment. 

## 7. Neuroinflammatory Reaction in VKC

The ocular surface is innervated by sensory and autonomic nerve fibers in both stroma and epithelial cells. Healthy human conjunctiva also expresses adrenergic and muscarinic receptors [81]. The concept of neuroinflammation has emerged in the last decades with the demonstration of a mutual interaction between nerves and immune cells [82]. Neuropeptides and neurotrophins represent the mediators of the neuroimmune reaction and play a pivotal role in the pathogenesis and development of allergic diseases [81,82,83]. Some evidence shows that neuromediators and neuropeptides, released by afferent nerve endings and by inflammatory and epithelial cells, participate in the inflammatory processes by inducing hyper-reactivity, vasodilatation, and plasma extravasation, as well as activation of immune cells, including mast cells, eosinophils, lymphocytes, and macrophages [84,85]. Specifically, it has been demonstrated that neuropeptides, including substance P, calcitonin-gene-related peptide (CGRP), neuropeptide Y (NPY), and vasoactive intestinal peptide (VIP), play a key role in allergic conjunctivitis [86]. In patients with VKC, a significant increase in tear and plasma levels of substance P was described when compared with normal controls [87,88]. Substance P release stimulates mucus secretion by goblet cells, histamine release by mast cells, Th2 cell differentiation, and recruitment and activation of eosinophils [84].

The demonstration of conjunctival hyper-reactivity in patients with VKC further supports the role of neurogenic inflammation in this condition. In fact, almost all patients with VKC complain of conjunctival hyper-reactivity symptoms, such as ocular itching, redness, and swelling in response to nonspecific stimuli, such as wind, cold and/or warm air, and sunlight exposure [37,85]. Hyper-reactivity represents a well-known clinical manifestation of allergic diseases, including asthma [85]. However, conjunctival hyper-reactivity not only is associated with inflammatory reaction but also involves neurogenic factors [82,88,89]. In patients with VKC, significant increase in plasma levels of nerve growth factor (NGF) has been described, and NGF levels were significantly correlated with the number of conjunctival mast cells [90,91,92]. In addition, the conjunctival expression of the NGF high-affinity receptor, TrKA, was found significantly increased in eosinophils and T-helper lymphocytes, infiltrating the stroma of VKC patients [91]. Thus, NGF may influence inflammatory reaction through binding to its own receptors expressed by mucosal resident cells and by B and T lymphocytes, mast cell, and eosinophils [93]. We recently demonstrated that NGF tear levels significantly increase after conjunctival allergen challenge in patients with allergic rhinoconjunctivitis (ARC) [82,89]. Interestingly, our data suggest that NGF is involved in the allergic reaction, also acting at the mucosal epithelium level, as demonstrated by the evidence of increased conjunctival expression of the low-affinity NGF receptor, p75NTR, in patients with ARC when compared with controls [89]. An in vitro study showed an increased expression of NGF and its receptor p75NTR in primary cultures of VKC-derived fibroblast (VKC-FB) supernatant. This study also showed that αSMA expression was enhanced by a specific neutralization of p75NTR, suggesting a specific role for NGF also in tissue remodeling in VKC [94]. Further studies are needed to investigate whether conjunctival p75NTR may represent a potential therapeutic target for ocular allergic diseases.

In addition, an autonomic dysfunction in VKC has been hypothesized based on evidence of alteration in the conjunctival expression of muscarinic and alpha 1 adrenergic receptors, as well as of neuropeptides, such as VIP and NGF, during active VKC inflammation [81].

Increasing understanding of the role of neuroinflammation in VKC is a promising research field that could provide novel therapeutic targets and improve the management of VKC.

## 8. Hormonal Influence in VKC

The higher incidence of VKC in boys as compared with girls, with a male-to-female ratio ranging from 4:1 to 2:1, and the spontaneous recovery after puberty strongly suggest a role of sex hormone influence in the development of VKC [2,3,95]. Bonini et al. reported that 2% of patients with VKC also had sex-hormone-related diseases, such as gynecomastia, mammary fibroadenoma, polycystic ovary syndrome, and adiposogenital dystrophy [2]. These findings suggest that sex hormones may be involved in the development of VKC and that the physiological changes of androgen pattern occurring at puberty may act on the immune system as protective factors and therefore induce recovery of VKC at puberty [96]. In fact, it has been shown that sex hormones influence the immune system, although their role in the homeostasis of immunity is not completely understood yet. Specifically, estrogens exert immune-enhancing activities, stimulating mast cell degranulation and allergic sensitization, while androgens may antagonize the production of Th2 cytokines and seem to act as anti-inflammatory hormones [96,97,98,99].

The first evidence of an involvement of sex hormones in VKC was provided by the demonstration of increased expression of estrogen and progesterone receptors in the conjunctiva of patients with VKC, mostly expressed by eosinophils [100]. Most recently, we described changes of circulating estrogen and androgen levels in VKC patients when compared with healthy subjects [97]. Specifically, even if within normal ranges, a significant decrease in circulating dihydrotestosterone (DHT), the most active androgen, was observed in VKC patients when compared with age- and sex-matched healthy subjects, suggesting that low levels of circulating androgens may have a role in the development of VKC. This hypothesis is supported by evidence of a normalization of DHT circulating levels in a group of adolescent patients with VKC in the remission phase [97].

Recently, adult-onset VKC was described, and an increase in ocular surface androgen receptor protein expression was reported in a group of patients with adult VKC when compared with childhood VKC, although within the normal ranges for age group [10,98].

A potential role of thyroid hormones in the pathogenesis of VKC has also been proposed. Specifically, some clinical studies reported that patients with VKC showed a high frequency of familial history of autoimmune disorders and antinuclear antibodies (ANA) and thyroid autoantibodies, suggesting a potential association of VKC with systemic autoimmune disorders [76,101]. Alterations of thyroid function, including hypothyroidism and positive thyroid autoantibodies, were also described in children with VKC [102].

Further investigations are needed to clarify the role of local alterations of sex hormone pathways and the involvement of thyroid function alterations in VKC and their potential use as therapeutic targets for ocular allergic diseases.

## 9. Conclusions

**Figure 3:**
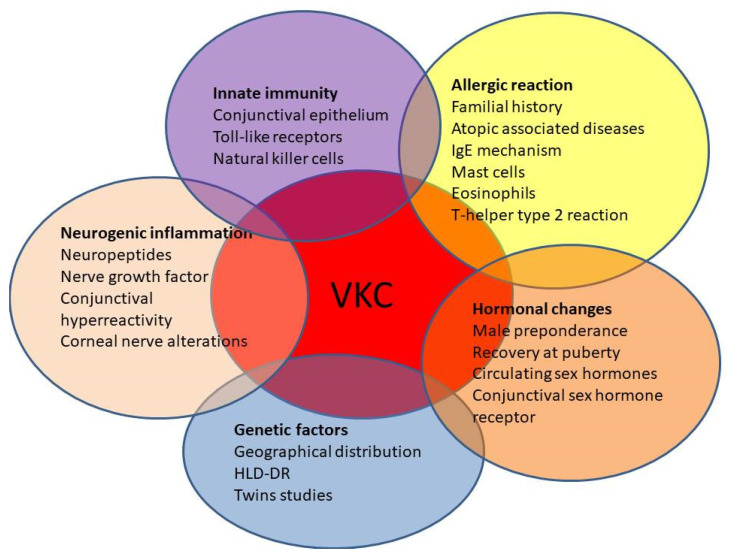
Allergic reaction represents the main pathogenic factor of VKC; however, several sources of evidence show that innate immunity and neuroinflammatory response, as well as genetic, hormonal, and environmental factors, also participate in the development and severity of VKC.

Despite increasing understanding of VKC in terms of clinical presentations and pathogenic mechanisms, several aspects of this challenging condition remain controversial. In fact, very little is known about the role of genetic susceptibility and the neuro–endocrine–immune interaction, which may participate in the pathogenesis of this disease (Figure 3).

Since acute exacerbations of VKC are successfully treated with short-term pulsed topical steroid administration, long-term management of this condition often requires the use of topical immunomodulatory drugs [4,16]. However, no specific pathogenic treatments are currently available for this sight-threatening disease. A better understanding of the pathogenesis of VKC is highly sought after in order to identify novel specific therapeutic targets to achieve a better control of this disease.

Several non-IgE-mediated mechanisms have been proposed for the pathogenesis of VKC, including alteration of innate immunity response, leading to an unbalance of Th1/Th2 reaction, changes of sex hormone pathways, and neuroinflammatory response. Increased efforts in research are needed to identify and develop novel molecules targeting specific pathogenic pathways in VKC, such as agents capable of modulating TLRs or addressing local hormonal changes. In addition, the exact role of ethnicity and its genetic relevance is still unknown; however, the identification of genetic factors influencing the development and/or severity of VKC should be useful to detect patients at higher risk of developing a severe form of the disease.
